# Supplementary material for: EMitool: Explainable Multi-Omics Integration for Disease Subtyping
Source: Int J Mol Sci. 2025 Apr 30;26(9):4268. doi: 10.3390/ijms26094268 (PMC12072579; doi:10.3390/ijms26094268)
Supplement: Supplementary file 1 [file ijms-26-04268-s001.zip › Supplementary Figures.pdf]

Supplementary Figures

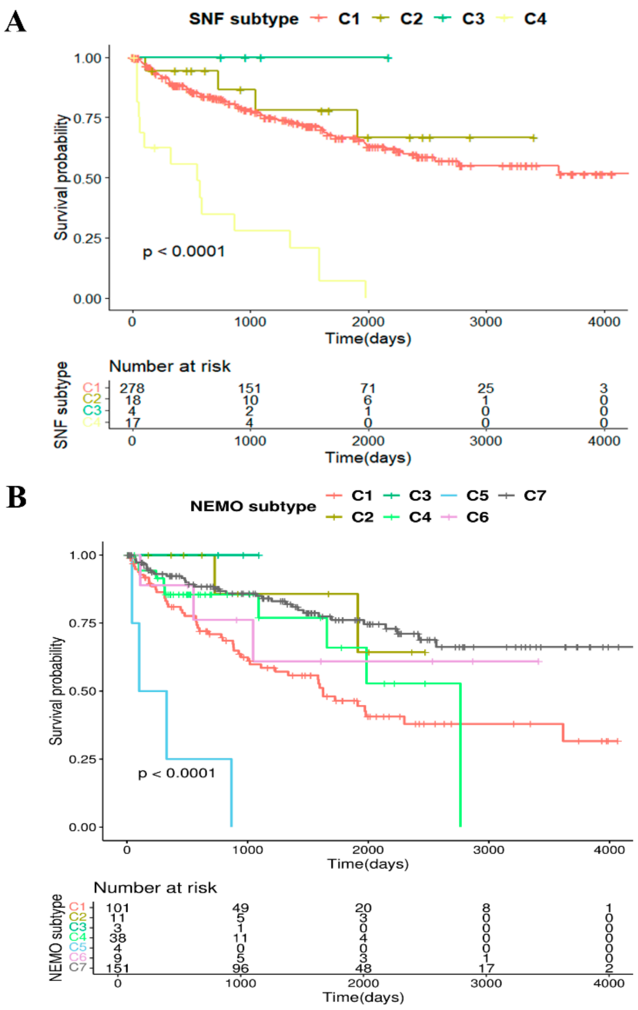

Figure S1. Kaplan–Meier survival curves of SNF subtypes and NEMO subtypes. (**A**) Kaplan–Meier survival curves after multi-omics integration-based subtyping of KIRC using the SNF tool. (**B**) Kaplan–Meier survival curves after multi-omics integration-based subtyping of KIRC using the NEMO tool.

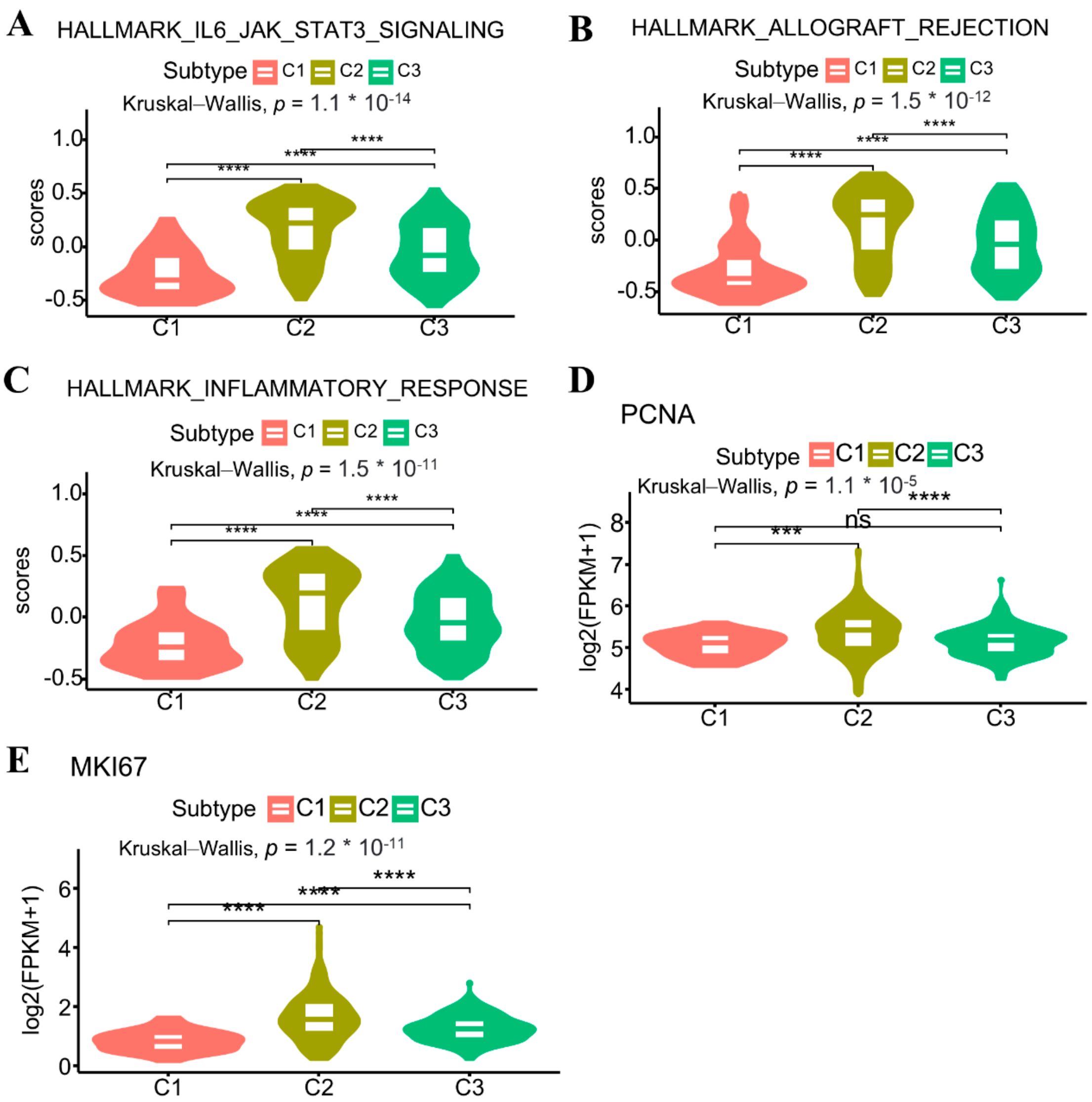

Figure S2. Single sample gene set variation analysis (ssGSVA) of cancer hallmark gene sets for different subtypes in KIRC. (**A**), (**B**), and (**C**) The violin plot shows the ssGSVA enrichment scores of different subtypes in HALLMARK_IL6_JAK_STAT3_SIGNALING, HALLMARK_ALLOGRAFT_REJECTION, and HALLMARK_INFLAMMATORY_RESPONSE (the Wilcoxon rank-sum test was used. *P < 0.05, **P < 0.01, ***P < 0.001, ****P < 0.0001). (**D**), (**E**) Differences in the expression levels of cell proliferation markers among different subtypes (the Wilcoxon rank-sum test was used. ns $p > 0.05$, *** $p < 0.001$, **** $p < 0.0001$).

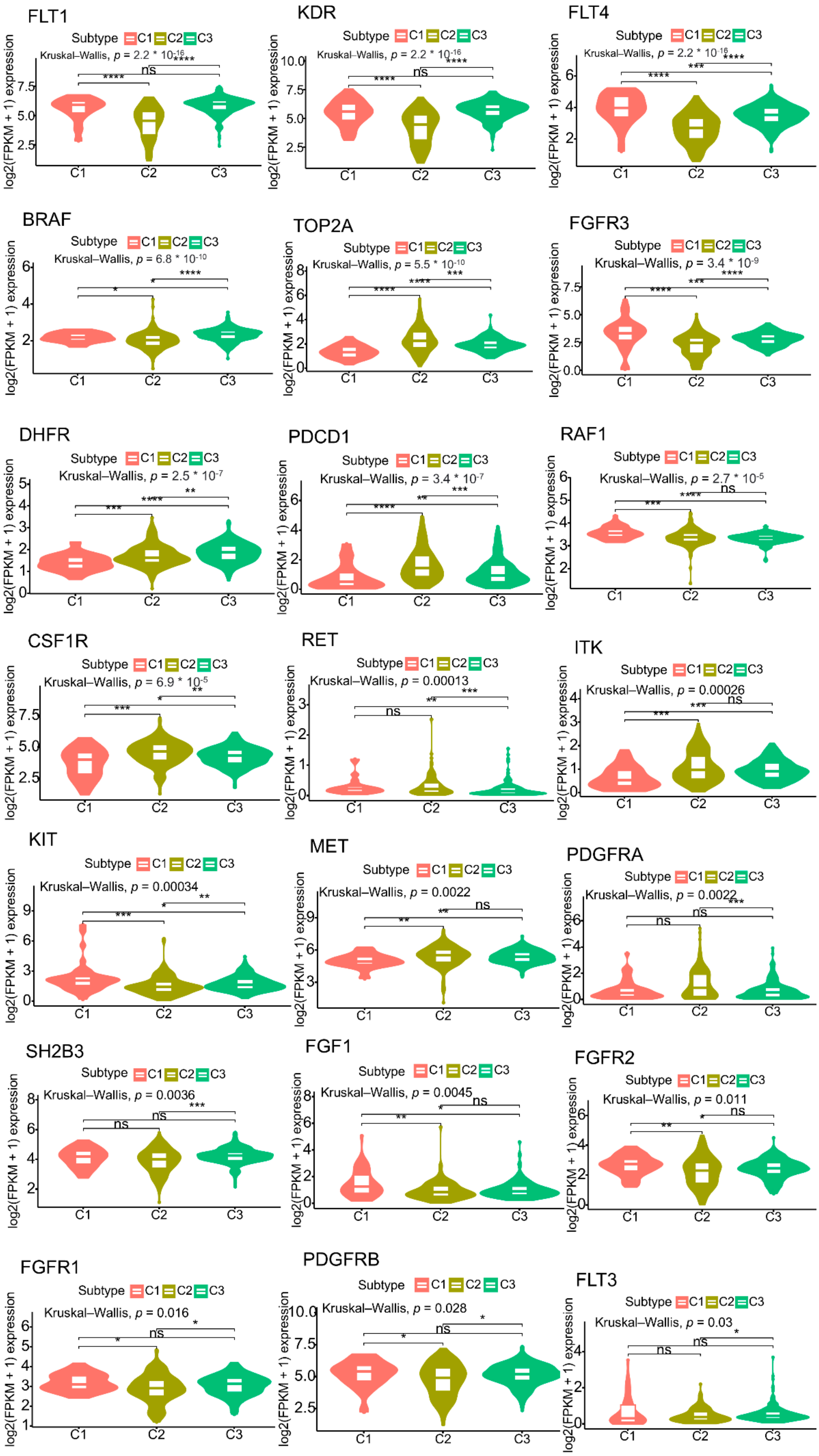
FLT1
Kruskal–Wallis, p = 2.2 * 10-16
KDR
Kruskal–Wallis, p = 2.2 * 10-16
FLT4
Kruskal–Wallis, p = 2.2 * 10-16
BRAF
Kruskal–Wallis, p = 6.8 * 10-10
TOP2A
Kruskal–Wallis, p = 5.5 * 10-10
FGFR3
Kruskal–Wallis, p = 3.4 * 10-9
DHFR
Kruskal–Wallis, p = 2.5 * 10-7
PDCD1
Kruskal–Wallis, p = 3.4 * 10-7
RAF1
Kruskal–Wallis, p = 2.7 * 10-5
CSF1R
Kruskal–Wallis, p = 6.9 * 10-5
RET
Kruskal–Wallis, p = 0.00013
ITK
Kruskal–Wallis, p = 0.00026
KIT
Kruskal–Wallis, p = 0.00034
MET
Kruskal–Wallis, p = 0.0022
PDGFRA
Kruskal–Wallis, p = 0.0022
SH2B3
Kruskal–Wallis, p = 0.0036
FGF1
Kruskal–Wallis, p = 0.0045
FGFR2
Kruskal–Wallis, p = 0.011
FGFR1
Kruskal–Wallis, p = 0.016
PDGFRB
Kruskal–Wallis, p = 0.028
FLT3
Kruskal–Wallis, p = 0.03
Subtype C1 C2 C3
log2(FPKM + 1) expression

Figure S3. Differences in the gene expression of known drug targets among different subtypes in KIRC (the Wilcoxon rank-sum test was used. ns $p > 0.05$, * $p < 0.05$, ** $p < 0.01$, *** $p < 0.001$, **** $p < 0.0001$).
