# Supplementary material for: EMitool: Explainable Multi-Omics Integration for Disease Subtyping
Source: Int J Mol Sci. 2025 Apr 30;26(9):4268. doi: 10.3390/ijms26094268 (PMC12072579; doi:10.3390/ijms26094268)
Supplement: Supplementary file 1 [file ijms-26-04268-s001.zip › Supplementary TableS1_S2_S5.pdf]

Supplementary Tables

Table S1: Analysis using Cox log-rank test p-values among multi-omics clustering algorithms. The red-colored p-value indicates that the p-value is less than 0.05.

Tumor	IntNMF	PINSPlus	LRAcluster	CIMLR	MANAclust	iclusterPlus	SNF	NEMO	EMitool
ACC	0.00034	0.013	0.000006	0.000013	0.0000001	0.00031	0.000009	0.000021	0.00000011
BLCA	0.0046	0.0034	0.053	0.0063	0.001	0.0032	0.032	0.00017	0.00031
BRCA	0.6	0.47	0.34	0.42	0.13	0.85	0.021	0.074	0.0065
CESC	0.87	0.95	0.39	0.57	0.0066	0.026	0.097	0.24	0.17
CHOL	0.9	0.25	0.16	0.31	0.74	0.091	0.49	0.4	0.073
COAD	0.7	0.69	0.44	0.84	0.47	0.22	0.038	0.11	0.019
DLBC	0.37	0.68	0.43	0.97	0.91	0.3	0.79	0.51	0.59
ESCA	0.35	0.014	0.017	0.69	0.25	0.52	0.3	0.41	0.015
HNSC	0.61	0.079	0.36	0.0091	0.11	0.49	0.02	0.0017	0.018
KICH	0.61	4.4E-14	1.6E-13	0.022	0.0000045	3E-13	0.00088	3.3E-11	2.6E-11
KIRC	0.00000042	0.00011	0.0018	0.0025	0.0000055	0.00053	1.1E-12	1.2E-09	0.0000011
KIRP	0.0000039	3.1E-19	0.24	0.0019	0.0000031	1.8E-15	4.4E-16	0.000013	0.0000037
LAML	0.14	0.46	0.94	0.0026	0.071	0.91	0.0041	0.078	0.0071
LGG	1.1E-08	1.6E-16	5.5E-15	3.4E-11	0.0000027	6.4E-25	5.1E-27	0.0000018	8.1E-10
LIHC	0.0041	0.051	0.044	0.000067	0.0074	0.00054	0.00072	0.00015	0.00004
LUAD	0.01	0.26	0.2	0.92	0.37	0.047	0.11	0.0000032	0.0068
LUSC	0.52	0.16	0.8	0.33	0.84	0.34	0.028	0.02	0.034
MESO	0.015	0.69	0.1	0.00085	0.000014	1.9E-11	1.7E-14	0.00039	0.00000031
OV	0.66	0.62	0.43	0.071	0.12	0.04	0.36	0.21	0.042
PAAD	0.00059	0.19	0.0047	0.0065	0.00058	0.0015	0.0017	0.00054	0.017
PCPG	0.51	0.036	0.081	0.46	0.0096	0.18	0.38	0.33	0.39
PRAD	0.33	0.39	0.63	0.31	0.53	0.47	0.29	0.34	0.59
SARC	0.17	0.34	0.34	0.034	0.057	0.094	0.0049	0.026	0.0066
SKCM	0.0000047	0.0044	0.064	0.0058	0.019	0.0059	0.0000029	0.0063	0.0066
STAD	0.031	0.7	0.14	0.5	0.41	0.44	0.58	0.56	0.24
TGCT	0.71	0.86	0.42	0.71	0.76	0.45	0.42	0.75	0.44
THCA	0.88	0.68	0.49	0.8	0.043	0.09	0.047	0.0073	0.0049
THYM	0.36	0.0082	0.0049	0.24	0.011	0.0036	0.028	0.0083	0.004
UCEC	0.0041	0.048	0.0019	0.062	0.078	0.0024	0.038	0.00017	0.1
UCS	0.92	0.26	0.66	0.85	0.82	0.76	0.23	0.97	0.33
UVM	0.33	0.0000021	0.025	0.00049	0.0000098	0.000002	0.0000028	0.00000018	0.00000025

Table S2: Analysis using Cox log-rank test p-values among single-omics clustering. The red-colored p-value indicates that the p-value is less than 0.05.

Tumor	mRNA	DNA methylation	miRNA	EMitool
ACC	0.00051	0.00039	0.00032	0.00000011
BLCA	0.00078	0.08	0.0011	0.00031
BRCA	0.18	0.1	0.88	0.0065
CESC	0.16	0.6	0.37	0.17
CHOL	0.51	1	0.8	0.073
COAD	0.27	0.67	0.28	0.019
DLBC	0.37	0.34	0.9	0.59
ESCA	0.49	0.25	0.079	0.015
HNSC	0.18	1	0.14	0.018
KICH	5.7E-11	0.00011	0.25	2.6E-11
KIRC	0.0001	0.00014	0.0037	0.0000011
KIRP	7.9E-09	4.6E-16	0.00059	0.0000037
LAML	0.084	0.16	0.014	0.0071
LGG	1E-28	2E-17	2.4E-12	8.1E-10
LIHC	0.000027	0.0062	0.00057	0.00004
LUAD	0.014	0.67	0.027	0.0068
LUSC	0.16	0.091	0.39	0.034

MESO	0.000027	0.023	0.0000029	0.00000031
OV	0.0014	0.45	0.066	0.042
PAAD	0.0042	0.039	0.0095	0.017
PCPG	0.25	0.4	0.13	0.39
PRAD	0.49	0.93	0.82	0.59
SARC	0.1	0.061	0.12	0.0066
SKCM	0.000044	0.0018	0.29	0.0066
STAD	0.38	0.013	0.18	0.24
TGCT	0.42	0.44	0.42	0.44
THCA	0.6	0.043	0.035	0.0049
THYM	0.054	0.11	0.018	0.004
UCEC	0.0067	0.006	0.078	0.1
UCS	0.25	0.77	0.39	0.33
UVM	0.0031	0.0000015	0.00000038	0.00000025

Table S5: Therapeutic drugs corresponding to target genes in KIRC subtypes. The mechanism of drug action based on the target is inhibitor or antagonist.

Target	Drug	Subtypes
FLT1	Sunitinib	C1, C3
	Sorafenib	C1, C3
	Pazopanib	C1, C3
	Axitinib	C1, C3
	Lenvatinib	C1, C3
KDR	Cabozantinib	C1, C3
	Sunitinib	C1, C3
	Sorafenib	C1, C3
	Pazopanib	C1, C3
	Axitinib	C1, C3
	Lenvatinib	C1, C3
FLT4	Sunitinib	C1
	Sorafenib	C1
	Axitinib	C1
	Lenvatinib	C1
BRAF	Sorafenib	C3
TOP2A	Doxorubicin	C2
FGFR3	Pazopanib	C1
	Lenvatinib	C1
DHFR	Methotrexate	C3
PDCD1	Nivolumab	C2
RAF1	Sorafenib	C1
CSF1R	Sunitinib	C2
RET	Cabozantinib	C1, C2
	Sorafenib	C1, C2
	Lenvatinib	C1, C2
ITK	Pazopanib	C2, C3
KIT	Sorafenib	C1
	Sunitinib	C1
	Pazopanib	C1
	Lenvatinib	C1
MET	Cabozantinib	C2, C3
	Sunitinib	C2, C3
PDGFRA	Sunitinib	C2
	Pazopanib	C2
	Lenvatinib	C2
SH2B3	Pazopanib	C3
FGF1	Pazopanib	C1
FGFR2	Lenvatinib	C1
FGFR1	Sorafenib	C1, C3
	Lenvatinib	C1, C3
PDGFRB	Sunitinib	C1, C3

	Sorafenib	C1, C3
	Pazopanib	C1, C3
FLT3	Sunitinib	C3
	Sorafenib	C3
