# Supplementary figures and images for: EMitool: Explainable Multi-Omics Integration for Disease Subtyping
Source: Int J Mol Sci. 2025 Apr 30;26(9):4268. doi: 10.3390/ijms26094268 (PMC12072579; doi:10.3390/ijms26094268)

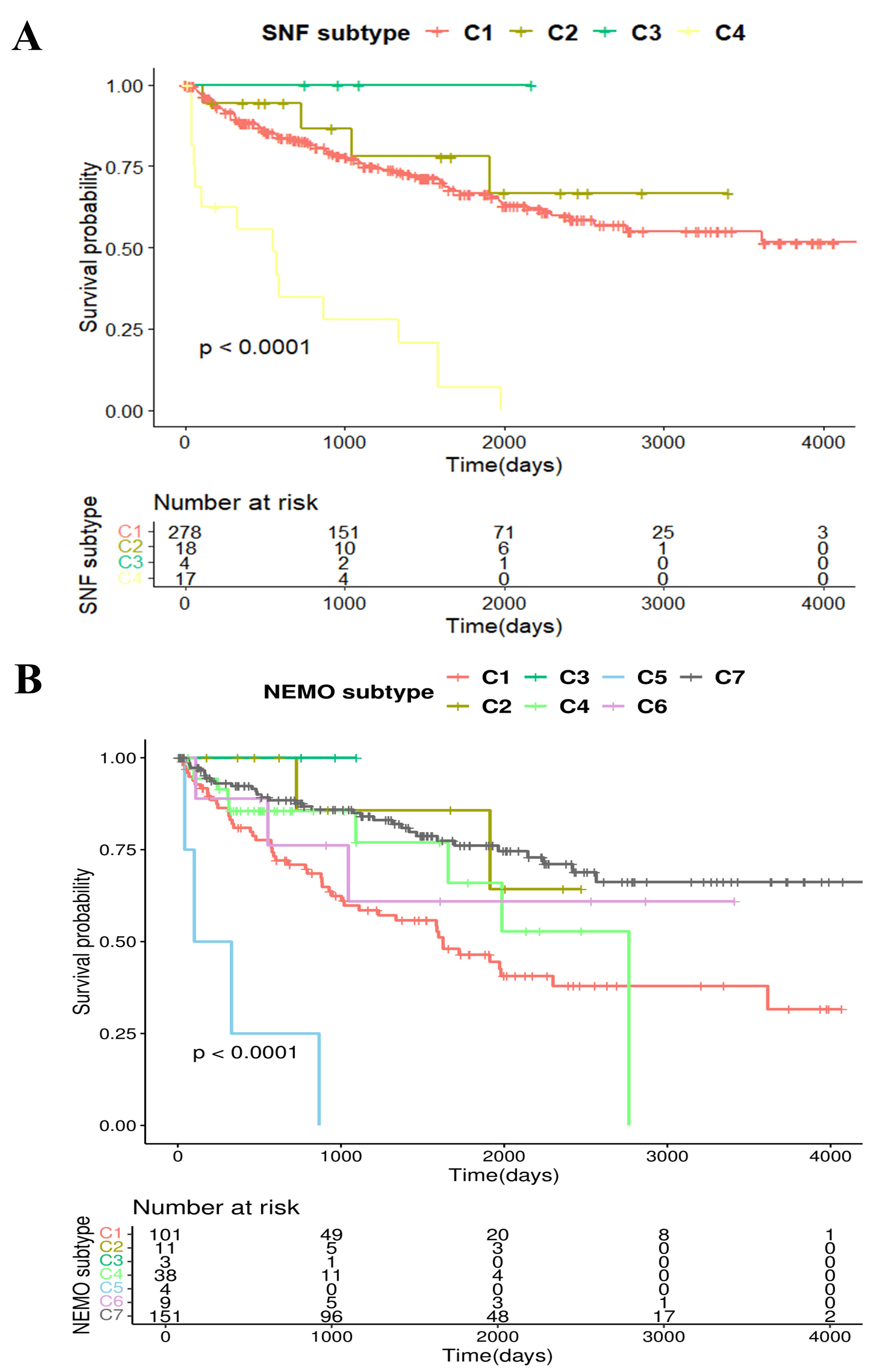

Supplement: Supplementary file 1 [file ijms-26-04268-s001.zip › FigureS1.tif]

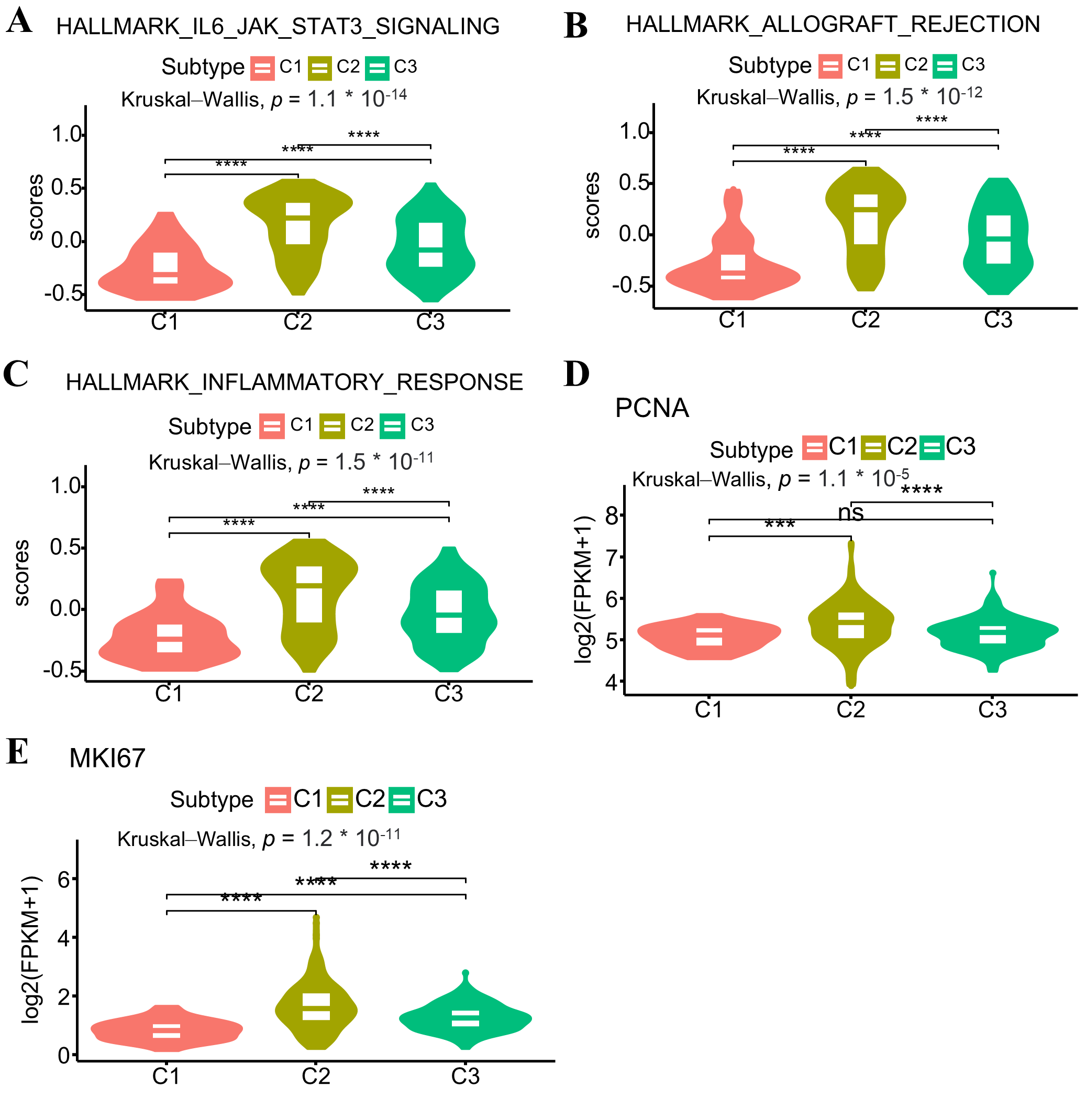

Supplement: Supplementary file 1 [file ijms-26-04268-s001.zip › FigureS2.tif]

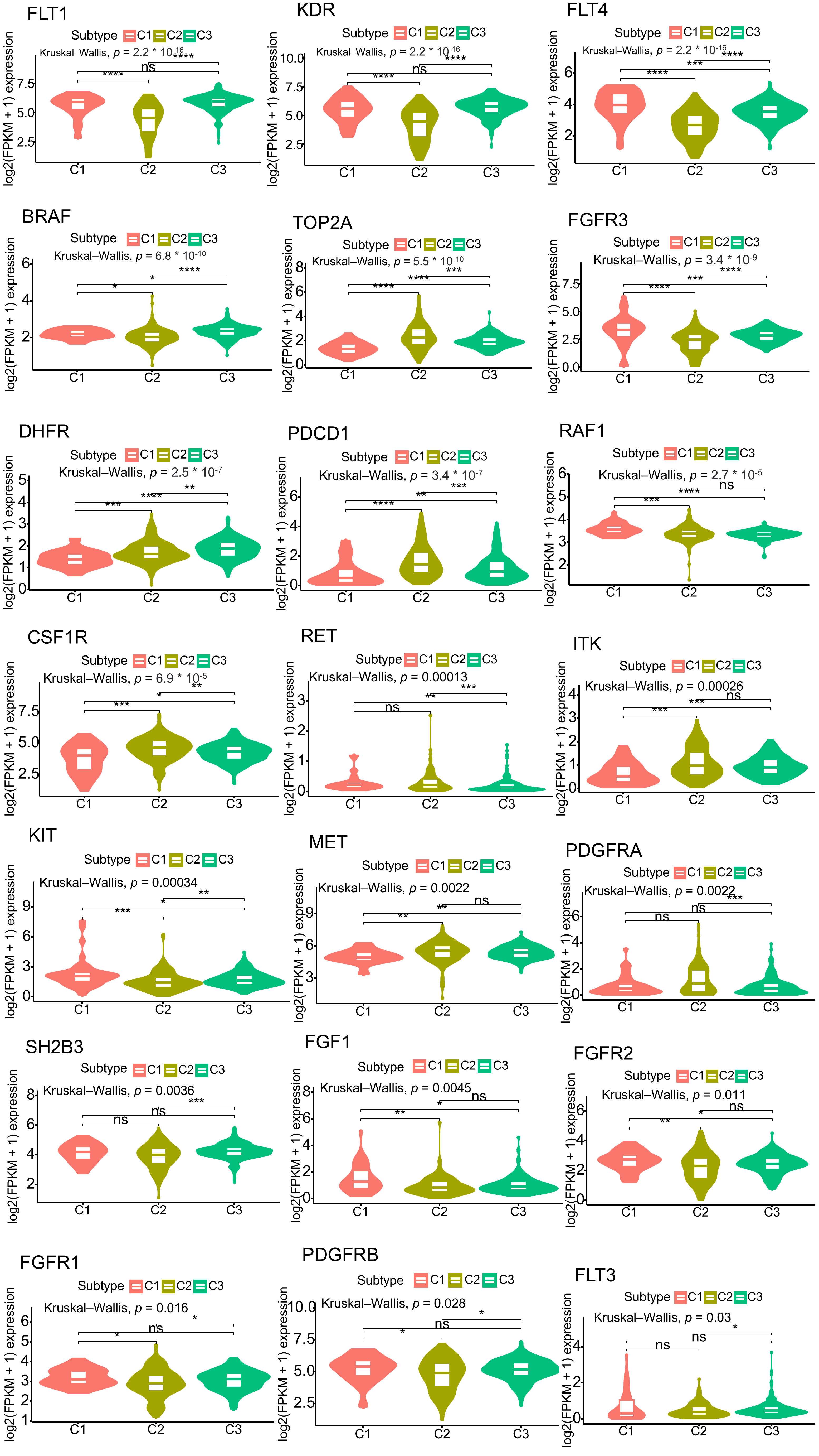

Supplement: Supplementary file 1 [file ijms-26-04268-s001.zip › FigureS3.tif]
